# Maternal Occupational Exposure to Noise during Pregnancy and Hearing Dysfunction in Children: A Nationwide Prospective Cohort Study in Sweden

**DOI:** 10.1289/ehp.1509874

**Published:** 2015-12-08

**Authors:** Jenny Selander, Maria Albin, Ulf Rosenhall, Lars Rylander, Marie Lewné, Per Gustavsson

**Affiliations:** 1 Institute of Environmental Medicine, Unit of Occupational Medicine, Karolinska Institutet, Stockholm, Sweden; 2 Centre for Occupational and Environmental Medicine at Stockholm County Council, Stockholm, Sweden; 3 Division of Occupational and Environmental Medicine, Lund University, Lund, Sweden; 4 Department of Audiology and Neurotology, Karolinska University Hospital, Stockholm, Sweden

## Abstract

**Background:**

Many women of childbearing age are occupationally active, which leads to a large number of pregnancies potentially exposed to occupational exposures. Occupational noise has been identified as a risk factor for hearing impairment in adults. However, very few studies have assessed the effect of occupational noise on the fetus.

**Objectives:**

The aim of this study was to investigate whether occupational exposure to noise during pregnancy is associated with hearing dysfunction in children.

**Methods:**

This population based cohort study included 1,422,333 single births in Sweden 1986–2008. Data on mothers’ occupation, smoking habits, age, ethnicity, body mass index, leave of absence, and socioeconomic factors were obtained from interviews performed by prenatal care unit staff at approximately 10 weeks of gestation and from national registers. Occupational noise exposure was classified by a job–exposure-matrix as < 75, 75–84, or ≥ 85 dBLAeq,8h. Diagnosed cases of hearing dysfunction (ICD-10 codes H90.3-7, 91.0, 91.2-3, 91.8, 93.1-2) were identified from a register of specialized medical care. Cox proportional hazards models were used to estimate associations.

**Results:**

In the full sample, containing a mixture of part-time and full-time workers during pregnancy, the adjusted HR for hearing dysfunction associated with maternal occupational noise exposure ≥ 85 vs. < 75 dBLAeq,8h was 1.27 (95% CI: 0.99, 1.64; 60 exposed cases). When restricted to children whose mothers worked full-time and had < 20 days leave of absence during pregnancy, the corresponding HR was 1.82 (95% CI: 1.08, 3.08; 14 exposed cases).

**Conclusions:**

This study showed an association between occupational noise exposure during pregnancy and hearing dysfunction in children. In view of mechanistic evidence and earlier indicative epidemiological and experimental findings, the results support that pregnant women should not be exposed to high levels of noise at work.

**Citation:**

Selander J, Albin M, Rosenhall U, Rylander L, Lewné M, Gustavsson P. 2016. Maternal occupational exposure to noise during pregnancy and hearing dysfunction in children: a nationwide prospective cohort study in Sweden. Environ Health Perspect 124:855–860; http://dx.doi.org/10.1289/ehp.1509874

## Introduction

A majority of women of child-bearing age in the industrialized part of the world are occupationally active today. In total, 73.7% of Swedish women of working age were employed in 2014 (OECD 2015). This leads to a high number of pregnancies potentially exposed to various occupational hazards. In Sweden, 15% of employed women report exposure to noise during at least one-quarter of the working day, so loud that they could not have a normal conversation (Arbetsmiljöverket 2012). Many studies have shown that occupational noise exposure causes hearing impairment in adults (Verbeek et al. 2012). However, very little is known about the association between occupational noise exposure during fetal life and hearing impairment in the child.

Sound is transmitted from the air over the abdominal wall and the uterus to the fetal head during pregnancy. The noise stimulates the inner ear through a soft tissue conduction route and could potentially affect the hearing of the fetus by damaging inner and outer hair cells within the cochlea, especially since the maturing cochlea is more sensitive to ototraumatic factors than the adult one (Brundin et al. 1989; Chordekar et al. 2012; Gerhardt and Abrams 2000). Both animal and human experimental studies show that the attenuation of noise through the passage of the abdominal wall and the uterus is strongly dependent on frequency. Although the fetus is well protected from high-frequency noise, low-frequency noise can even be amplified during the passage of the abdominal wall and the amniotic fluid (Chordekar et al. 2012; Gerhardt and Abrams 2000; Saunders and Chen 1982). In addition, animal experimental studies indicate that intense and sustained noise exposure during pregnancy can induce hearing dysfunction in the offspring in guinea pigs (Cook et al. 1982) and in sheep (Gerhardt and Abrams 2000; Griffiths et al. 1994; Huang et al. 1997). Three previous small epidemiological cross-sectional studies have investigated occupational noise exposure during pregnancy and hearing impairment in children, including a study of 131 4- to 10-year-old children in Quebec, Canada; a study of 75 children 10–14 years old in France; and a study of 80 children 0–6 years old in Brazil (Daniel and Laciak 1982; Lalande et al. 1986; Rocha et al. 2007). Although the studies conducted in Canada and France reported evidence supporting the hypothesis of an association between occupational noise exposures during pregnancy and hearing loss in children, the Brazilian study did not report an association. However, the studies were all based on small samples and therefore had low precision. The Brazilian study presented unspecific inclusion criteria, the French study lacked a comparison group, and the Canadian study had only two cases in the comparison group.

The aim of this study was to investigate if occupational exposure to noise during pregnancy is associated with an increased risk of hearing dysfunction in children in a population-based nationwide study with individual data on occupation, hearing dysfunction, and potential confounding factors.

## Methods

The FENIX (fetal noise exposure) study is a nationwide prospective cohort study based on births in Sweden between 1986 and 2008.

### Data Sources

Information on job title and if the woman was working full time, part time, or not working at all was collected at the registration interview for prenatal care (gestational week 10) together with background characteristics such as maternal age, smoking habits, family structure, and nationality. It was entered in the Medical Birth Register, together with information from records from the maternity wards regarding parity, gender, and date of birth. The Swedish Medical Birth Register includes 98–99% of all children born in Sweden (Socialstyrelsen 2002).

Information on hearing dysfunction was retrieved from the patient register, which includes diagnoses from all outpatient clinics for specialist care in Sweden from 2003 and onwards. The overall coverage in the patient register is approximately 80% (Socialstyrelsen 2013), but has not been specifically evaluated for hearing dysfunction.

Data on the mothers highest completed education at the year of the child’s birth was gathered through the population-based nationwide register (LISA) holding information on all Swedish citizens at > 16 years of age (Statistiska centralbyrån 2011).

Individual information on leave of absence during each pregnancy was retrieved from the Social Insurance MIDAS database and includes information on days of sick-leave and parental leave for the time period 1986–2008. The register has a complete coverage of long-term leave of absence, since every citizen needs to register sick leave or parental leave to receive payment from the Social Insurance Agency, but it does not cover short-term sick leave (≤ 14 cohesive days). However, parental leave and special sick leave related to the pregnancy is reported from day 1 (Försäkringskassan 2011).

The registers were matched with the personal identification number, unique to every Swedish citizen.

### Exposure

**Figure 1:**
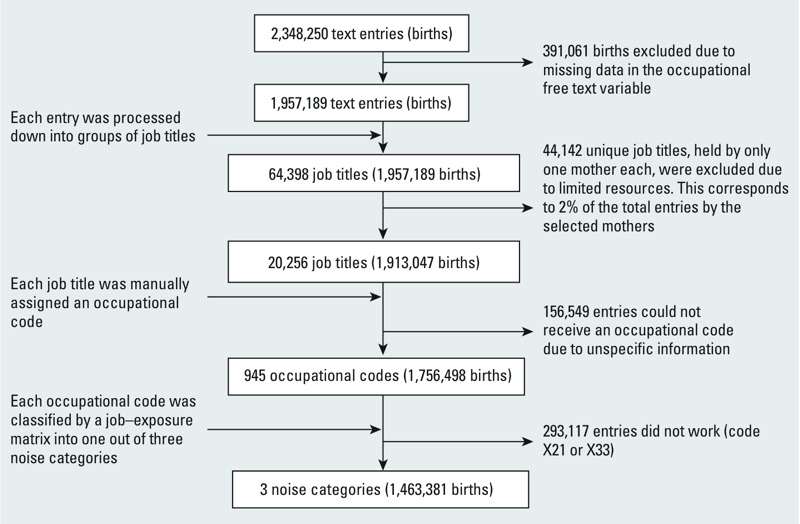
Schematic figure showing the process of classifying an occupational free text variable into one out of three noise categories < 75 dBA, 75–84 dBA, ≥ 85 dBA.

Job titles were recorded at the registration interview held at the prenatal care facilities in Sweden. The occupation was recorded as free text (not coded) in the Medical Birth Register. Out of the 2,348,250 births eligible for the study (children born between 1986 and 2008 in Sweden), 1,957,189 had data on occupation. The entries were processed down into 64,398 unique occupational titles by removing characters other than letters, by harmonizing abbreviations, and correcting spelling errors. There were approximately 44,142 unique titles each held by only one mother in the cohort; these were excluded from the study because of limited resources for coding of occupations, leaving 20,256 job titles held by at least two mothers and covering 98% of the occupational titles. These were coded manually by an occupational hygienist and a safety engineer into a 6-digit AMSYK/SSYK-code based on ISCO-88 (International Standard Classification of Occupations) [International Labour Organization (ILO) 1990] (Figure 1). Occupations that were difficult to assess were discussed among the two assessors until consensus was reached. However, 156,549 entries could not be assigned an occupational code because the information was too unspecific. In addition, 293,117 entries contained nonworking groups such as students, unemployed persons, housewives, and refugees (AMSYK-code X33 and X21). Because we focused in this study on occupational exposures, these mothers were excluded, leaving 1,463,381 births in the study classified into three noise categories. The study was further restricted to single births, and the final sample consisted of 1,422,333 births.

The validated job–exposure matrix was constructed before this study and is presented in detail elsewhere (Sjöström et al. 2013). Briefly, the job–exposure matrix contains 321 job families and specifies the annual average of the daily 8-hr noise exposure levels in three exposures categories [< 75, 75–84, ≥ 85 dBA (A-weighted decibels)] in 5-year calendar bands from 1970 to 2004. The noise exposure information used for the job–exposure matrix derives from measurement reports collected from occupational medicine clinics, occupational health services, and large companies all over Sweden. The highest occupational noise category, > 85 dBA, is based on the occupational exposure limit for noise in Sweden; the lower interval, < 75 dBA, includes occupations without a dominating noise source.

The occupational codes in this study were linked to the job–exposure matrix that classified the 8-hr average exposure (L_Aeq8h_) of each occupation into three categories: < 75 dBA, 75–84 dBA, ≥ 85 dBA (Figure 1). The job–exposure matrix was matched in 5-year calendar bands to each occupational code so that the exposure was applied to births occurring within that time frame. It was not possible to classify exposure to low-frequency noise (dBC) separately due to lack of frequency-specific exposure data.

### Outcome

We retrieved information on every child born during the period 1986–2008 who had been diagnosed in 2003–2008 with types of hearing dysfunctions that can be related to noise exposure during pregnancy in the patient register [*International Classification of Diseases, 10th Revision* (ICD-10)]: other diseases of inner ear (codes H83.3, H83.8), conductive mixed with sensorineural hearing loss and sensorineural hearing loss (H90.3-7), other hearing loss (H91.0, H91.2-3, H91.8), and other disorders of the ear, not elsewhere classified (including tinnitus) (H93.1, H93.2). This selection is based on the well-established concept that noise-induced hearing loss is of the sensorineural type, caused primarily by a cochlear damage (Hackney and Furness 2007). Diagnoses indicating middle ear problems or conductive hearing loss have not been included because they are not related to noise exposure. The total sample of all selected hearing disorders was analyzed together as one group. In addition, the two main diagnosis types—sensorineural hearing loss and tinnitus—that accounted for most of the cases were large enough to be analyzed separately. In these two subgroups, the same child could be included as a case in both the sensorineural hearing loss analysis and the tinnitus analysis if s/he had been diagnosed with both disorders. In the main analysis of all cases combined, only the first diagnosis was counted.

### Data Analysis

Information on diagnoses was available only for the time period 2003 to 2008, whereas the cohort included all births from 1986. Thus, the chance of having a hearing dysfunction diagnosis varies with year of birth, and all analyses were therefore adjusted for birth year (22 categories).

All analyses were performed using Cox proportional hazards regression with child’s age as the underlying time scale. Person-years for each child were calculated from the child’s birth to the age at first diagnosis or age at the end of follow-up (31 December 2008), whichever came first. Potential confounders were identified through a review of previous studies on pregnancy outcomes and occupational exposures, by observing the effect each potential confounder had on the association between exposure and outcome and by placing the potential confounders in a causal web. All variables initially assessed were selected as covariates and are presented in Table 1: mother’s age (quartiles), smoking (three categories: not smoking, 1–9 cigarettes/day, ≥ 10 cigarettes/day), highest completed education (four categories: pre-high school, high school, university < 3 years, university ≥ 3 years), nationality (four categories: Swedish, other European country, outside Europe, unknown), family structure (dichotomous: married/living together with the father, single mother), child’s sex (dichotomous: male/female), birth year (quartiles) and parity (three categories: 1, 2, or ≥ 3). Complete case analyses were performed and observations with missing data for any model covariates were thereby excluded when estimating adjusted hazard ratios (HRs).

**Table 1:** Baseline characteristics of the study participants and maternal occupational noise exposure during pregnancy [*n* (%)].

Characteristics**	Occupational noise exposure^*a*^		
	< 75 dBA	75–84 dBA	≥ 85 dBA
*n* total sample (1,422,333)	1,126,356	290,071	5,906
Mother’s characteristics
Age (quartiles)
≤ 24 years	175,994 (15.6)	59,519 (20.5)	1,498 (25.4)
25–28 years	311,072 (27.6)	86,938 (30.0)	1,671 (28.3)
29–32 years	336,334 (29.9)	79,124 (27.3)	1,430 (24.2)
≥ 33 years	302,956 (26.9)	64,490 (22.2)	1,307 (22.1)
Missing	0 (0)	0 (0)	0 (0)
Smoking
Nonsmokers	938,088 (85.9)	225,991 (80.5)	4,434 (77.3)
Smokers, 1–9 cig per day	105,229 (9.6)	34,062 (12.1)	756 (13.2)
Smokers, ≥ 10 cig per day	49,190 (4.5)	20,549 (7.3)	545 (9.5)
Missing	33,849 (3.0)	9,469 (3.3)	171 (2.9)
Highest completed educational level
Pre-high school	96,232 (8.7)	52,666 (18.7)	1,222 (21.3)
High school	610,581 (54.9)	133,051 (47.2)	3,593 (62.7)
University < 3 years	151,663 (13.6)	70,206 (24.9)	323 (5.6)
University ≥ 3 years	254,089 (22.8)	25,840 (9.2)	593 (10.4)
Missing	13,791 (1.2)	8,308 (2.9)	175 (3.0)
Nationality
Swedish	1,047,984 (93.1)	257,149 (88.7)	5,348 (90.6)
Other European country	39,513 (3.5)	15,089 (5.2)	314 (5.3)
Outside Europe	16,682 (1.5)	9,445 (3.3)	99 (1.7)
Unknown	22,030 (2.0)	8,306 (2.9)	144 (2.4)
Missing	147 (0)	82 (0)	1 (0)
Working at beginning of pregnancy^*b*^
No	59,881 (5.9)	14,312 (5.5)	292 (5.4)
Part time	377,291 (37.2)	91,079 (35.2)	1,251 (23.3)
Full time	576,487 (56.9)	153,186 (59.2)	3,824 (71.3)
Missing	112,697 (10.0)	31,494 (10.9)	539 (9.1)
Leave of absence^*c*^
< 20 days	515,358 (45.8)	106,403 (36.7)	1,845 (31.2)
20–153 days	513,628 (45.6)	151,814 (52.3)	3,214 (54.4)
> 153 days	97,370 (8.6)	31,854 (11.0)	847 (14.3)
Missing	0 (0)	0 (0)	0 (0)
Family structure
Married/living together with the father	1,052,832 (96.4)	266,783 (95.1)	5,490 (96.0)
Missing	34,455 (3.1)	9,563 (3.3)	186 (3.1)
Child’s characteristics
Sex
Male	578,886 (51.4)	149,087 (51.4)	2,986 (50.6)
Missing	0 (0)	0 (0)	0 (0)
Parity
1	500,459 (44.4)	119,747 (41.3)	2,596 (44.0)
2	413,151 (36.7)	103,068 (35.5)	2,035 (34.5)
≥ 3	212,746 (18.9)	67,256 (23.2)	1,275 (21.6)
Missing	0 (0)	0 (0)	0 (0)
Birth year
1986–1990	258,479 (23.0)	86,280 (29.7)	1,439 (24.4)
1991–1996	318,963 (28.3)	87,659 (30.2)	1,871 (31.7)
1997–2002	245,798 (21.8)	57,062 (19.7)	1,315 (22.3)
2003–2008	303,116 (26.9)	59,070 (20.4)	1,281 (21.7)
Missing	0 (0)	0 (0)	0 (0)
^***a***^Occupational noise exposure estimated through a job–exposure matrix based on measurements at several work sites, subdividing the mother’s occupation registered at the beginning of the pregnancy into three noise categories. ^***b***^Includes mothers who had reported an occupation at the beginning of pregnancy (gestational week 10), and divides them according to their answer on the separate presence-at-work-question (Do you work right now? no/part time/full time). ^***c***^Number of days of leave of absence due to sick leave or parental leave reported to the Social Insurance Agency register during pregnancy, including a summation of all days between the conception date (birthdate – days of gestation = conception date) and the birth date. Cut-off was at the median level 20 days and at 153 days of absence, equivalent to the 90th percentile.			

All analyses were restricted to single births and to employed women by excluding women who at the beginning of pregnancy (interview gestational week 10) said that they were students, unemployed, housewives, refugees, or in other nonworking categories (AMSYK-code X33 and X21) during pregnancy. Women who in gestational week 10 stated an occupation that could be coded into occupational codes were included in the study. The presence of the pregnant worker at the workplace was then evaluated and certain analyses were restricted regarding work participation by combining the answers to the question on current degree (work full time, part time, or not at all right now) of occupational activity at beginning of pregnancy (interview at gestational week 10) with the registry data on days of absence (sick leave, parental leave, pregnancy benefit) during the whole pregnancy. This was done by dividing the cohort into three categories: absent from work, part-time work, and full-time work. Absent from work included mothers who stated an occupation but also stated that they did not work at the registration interview in gestational week 10 or mothers who stated an occupation but had more than 153 days (90th percentile) of leave of absence from work during pregnancy. Part-time workers were defined as mothers who stated at the registration interview that they worked part-time or had between 20 (median) and 153 days (90th percentile) of leave of absence during pregnancy. Full-time workers were defined as mothers stating at the registration interview that they worked full-time at the beginning of pregnancy and had < 20 days (median) of sick leave during pregnancy.

Statistical significance was determined by 95% confidence intervals (CIs). All analyses were performed with STATA SE 12 (StataCorp), and the study was approved by the regional ethics committee in Stockholm.

## Results

The study included 1,422,333 single births. Of these, 1,320,195 had complete data for the covariates of the adjusted model. In total, 12,668 cases of hearing dysfunction were identified and included in the study; other diseases of inner ear, 133 cases (ICD-10 codes H83.3, H83.8), conductive mixed with sensorineural hearing loss and sensorineural hearing loss, 8,696 cases (H90.3-7), other hearing loss, 917 cases (H91.0, H91.2-3, H91.8), and other disorders of the ear, not elsewhere classified (including tinnitus), 3,637 cases (H93.1, H93.2).

Background characteristics are presented in Table 1. Compared with less-exposed mothers (< 75 dBA), highly exposed mothers (≥ 85 dBA) tended to be younger (25% vs. 16% ≤ 24 years), less educated (21% vs. 8.7% < high school), and more likely to smoke during pregnancy (23% vs. 14%).The percentage of missing data in the background characteristics was low (0–10%). The three most frequent jobs in the high exposure group (≥ 85 dBA) were musicians (15%), carpenters, and wood workers (15%), and butchers (12%).

An increased risk of hearing dysfunction among children was indicated after exposure to occupational noise during pregnancy, Table 2. For all cases combined, adjusted HRs for 75–84 dBA and ≥ 85 dBA compared with < 75 dBA were 1.05 (95% CI: 1.00, 1.10) and 1.27 (95% CI: 0.99, 1.64), respectively. Corresponding estimates were similar for sensorineural hearing loss (HR = 1.08; 95% CI: 1.03, 1.15 and HR = 1.26; 95% CI: 0.93, 1.70). For tinnitus, moderate noise was not associated with an increased risk (HR = 0.99; 95% CI: 0.90, 1.08), but the HR for high noise ≥ 85 dBA was similar to the other outcomes (HR = 1.25; 95% CI: 0.74, 2.12). There were 60, 42, and 14 highly exposed cases for all hearing dysfunction, sensorineural hearing loss, and tinnitus, respectively.

**Table 2:** Hazard ratios (HRs) for maternal occupational noise exposure during pregnancy and hearing dysfunction in 1,422,333 children (1,320,195 children in the adjusted analyses).

Occupational noise exposure (dBA)^*a*^	Hearing dysfunction, all^*b*^			Sensorineural hearing loss^*c*^			Tinnitus^*d*^		
	*n* cases (crude/adjusted)	Crude^*e*^[HR (95% CI)]	Adjusted^*f*^ [HR (95% CI)]	*n* cases (crude/adjusted)	Crude^*e*^ [HR (95% CI)]	Adjusted^*f*^ [HR (95% CI)]	*n* cases (crude/adjusted)	Crude^*e*^ [HR (95% CI)]	Adjusted^*f*^ [HR (95% CI)]
< 75 dBA	9,813/9,001	1.00	1.00	6,688/6,176	1.00	1.00	2,548/2,285	1.00	1.00
75–84 dBA	2,790/2,545	0.96 (0.92, 1.00)	1.05 (1.00, 1.10)	1,962/1782	1.01 (0.96, 1.06)	1.08 (1.03, 1.15)	706/645	0.89 (0.82, 0.97)	0.99 (0.90, 1.08)
≥ 85 dBA	65/60	1.20 (0.94, 1.53)	1.27 (0.99, 1.64)	46/42	1.26 (0.94, 1.68)	1.26 (0.93, 1.70)	16/14	1.10 (0.67, 1.80)	1.25 (0.74, 2.12)
^***a***^Occupational noise exposure estimated through a job–exposure matrix based on measurements at several work sites, classifying the mother’s occupation registered at the beginning of the pregnancy (gestational week 10) into one of three noise categories. ^***b***^Hearing dysfunction all selected diagnoses, includes other diseases of inner ear (ICD-10 codes H83.3, H83.8), sensorineural hearing loss and sensorineural mixed with conductive hearing loss (H90.3-7), other hearing loss (H91.0, H91.2-3, H91.8), and other disorders of ear, not elsewhere classified (H93.1, H93.2). ^***c***^Sensorineural hearing loss includes sensorineural hearing loss and mixed conductive and sensorineural ICD-10: H90.3-7. ^***d***^Tinnitus includes ICD10: H93.1. ^***e***^Crude analyses were restricted to all single births between 1986 and 2008 and excluding mothers in nonworking categories (students, house wives, unemployed, refugees). ^***f***^Analyses adjusted for mother’s age, smoking, education, family structure, and nationality and child’s sex, birth year, and parity. Including all single births between 1986 and 2008 and excluding mothers in nonworking categories (students, housewives, refugees).									

The risk of hearing dysfunction among children in relation to occupational noise during pregnancy, subdivided by presence at work during pregnancy, is presented in Table 3. The association was strongest when analyses were restricted to mothers who worked full time during pregnancy with < 20 days of absence (362,572 total, 343,712 with complete data). The adjusted HR for occupational noise exposure ≥ 85 versus < 75 dBA was 1.82 (95% CI: 1.08, 3.08) based on 14 exposed cases and 2,222 cases with low exposure. In contrast, corresponding HRs were 1.25 (95% CI: 0.91, 1.71) for high exposure among mothers classified as working part time (37 exposed and 5,243 low-exposed cases) and 0.74 (95% CI: 0.35, 1.56) for women who had > 153 days (90th percentile) of absence from work during pregnancy or who were not working at the time of the registration interview (7 exposed and 1,116 unexposed cases).

**Table 3:** HRs in three separate analyses for maternal occupational noise exposure during pregnancy and hearing dysfunction,*^a^* dividing employed mothers by work participation during pregnancy.

Occupational noise exposure (dBA)^*b*^	Absent from work^*c*^			Working part time^*d*^			Working full time^*e*^		
	*n* cases (crude/adjusted)	Crude *n* = 181,170 [HR (95% CI)]	Adjusted^*f*^ *n* = 164,598 [HR (95% CI)]	*n* cases (crude/adjusted)	Crude *n* = 819,489 [HR (95% CI)]	Adjusted^*f*^ *n* = 760,049 [HR (95% CI)]	*n* cases (crude/adjusted)	Crude *n* = 362,572 [HR (95% CI)]	Adjusted^*f*^ *n* = 343,712 [HR (95% CI)]
< 75	1,223/1,116	1.00	1.00	5,749/5,243	1.00	1.00	2,363/2,222	1.00	1.00
75–84	410/368	0.89 (0.79, 1.00)	0.99 (0.88, 1.12)	1,804/1,645	1.00 (0.95, 1.06)	1.08 (1.02, 1.14)	447/450	0.97 (0.87, 1.07)	1.04 (0.93, 1.16)
≥ 85	7/7	0.60 (0.29, 1.26)	0.74 (0.35, 1.56)	40/37	1.26 (0.92, 1.71)	1.25 (0.91, 1.71)	16/14	1.91 (1.17, 3.13)	1.82 (1.08, 3.08)
^***a***^Hearing dysfunction (all selected diagnoses), includes other diseases of inner ear (ICD-10 codes H83.3, H83.8), sensorineural hearing loss and sensorineural mixed with conductive hearing loss (H90.3-7), other hearing loss (H91.0, H91.2-3, H91.8), and other disorders of ear, not elsewhere classified (H93.1, H93.2). ^***b***^Occupational noise exposure estimated through a job–exposure matrix based on measurements at several work sites, dividing the mother’s occupation registered at the beginning of the pregnancy (gestational week 10) into three noise categories. ^***c***^Employed mothers with an absence of > 153 days (90th percentile) during pregnancy or who reported “not working at the moment” during the registration interview at the prenatal care service in the beginning of pregnancy (gestational week 10). ^***d***^Mothers who reported that they worked part time at the beginning of pregnancy (gestational week 10) or had > 20 days (50th percentile) and < 153 days (90th percentile) absence from work during pregnancy. ^***e***^Mothers who reported that they worked full time at the beginning of pregnancy (gestational week 10) and had < 20 days (50th percentile) absence from work during pregnancy. ^***f***^Analyses adjusted for mother’s age, smoking, education, family structure, and nationality and child’s sex, birth year, and parity. Including all single births between 1986 and 2008 and excluding mothers in nonworking categories (students, housewives, refugees).									

## Discussion

An association between maternal occupational noise exposure > 85 dBA during pregnancy and hearing dysfunction among children was indicated. The association was more pronounced when restricting the study to full-time working mothers with < 20 days’ leave of absence during pregnancy. The results are in line with two previous epidemiological cross-sectional studies on occupational noise exposure and hearing impairment that reported an increased risk of hearing impairment after exposure to high occupational noise ≥ 85 dBA during pregnancy (Daniel and Laciak 1982; Lalande et al. 1986). In addition, animal studies have shown an association between noise exposure during pregnancy and hearing loss in offspring (Cook et al. 1982; Griffiths et al. 1994).

In the human fetus as well as in precocial mammals, the auditory system is functional well before birth (Armitage et al. 1980). The peripheral auditory system, including the cochlea, has an adult anatomical appearance by the 20th gestational week (Pujol et al. 1990), and the fetus responds to auditory stimulation at that time (Hepper and Shahidullah 1994). However, the maturing cochlea is more sensitive to ototraumatic factors than the fully developed cochlea (Saunders and Chen 1982). The sound transmission mechanisms after birth are not applicable to the fetal cochlea. Instead, the sound stimulates the inner ear through a soft-tissue conduction route (Chordekar et al. 2012). The sound passes through the abdominal wall, the uterine wall, and the amniotic fluid before reaching the fetal head, where a bone conduction route through the cartilaginous fetal skull bones is activated. Experimental studies both in sheep and humans show that mid- and high-frequency sounds are attenuated by the abdominal soft tissues (> 20 dB reduction) (Armitage et al. 1980; Gerhardt et al. 1990, 2000), but low-frequency sounds are not (0–5 dB reduction) (Gerhardt et al. 1990; Querleu et al. 1989; Sohmer and Freeman 2001); there is even one report showing that low frequency sound can be amplified (Richards et al. 1992). Impulse noise of 169 dB peak SPL (sound pressure level) in air was attenuated fairly modestly to 161 dB peak SPL near the head of a fetal sheep, indicating very little protection for the fetus against high-level impulse sounds (Gerhardt et al. 2000).

The fetus is exposed to sounds not only from external sources, but also from the mother’s own body. However, the natural intrauterine sound level consist mainly of very low-frequency noise (Benzaquen et al. 1990), and because sound consists of a mixture of frequencies (between 20 Hz to 20,000 Hz), the total noise exposure *in utero* to a large extent depends on the sound exposure outside the abdomen.

It was not possible to assess the risk associated with noise exposure at different stages in pregnancy in this study. Experimental studies on pregnant guinea pigs and sheep that also have a fully developed auditory system *in utero* show a risk of adverse effects of noise on the fetal hearing during the later gestational ages, corresponding to the last trimester of pregnancy in humans (Cook et al. 1982; Gerhardt et al. 1999). However, these studies do not provide any information about the risk at different stages of pregnancy. Even though the auditory system is not fully developed until the 20th gestational week in humans, there is a possibility of adverse influence of noise on the hearing even during the embryonic period in the first trimester, when the auditory system is developing. The otocyst embryonic stem cells are able to produce hair cell–like cells (Woods et al. 2004), and it can be speculated that these progenitor cells might be able to react in the event of loud sounds. Isolated adult mammalian cochlear outer hair cells respond with a change in length when subjected to sound stimulation (Canlon et al. 1988), and each cell has a sharply toned frequency response (Brundin et al. 1989). If the progenitor hair cell possesses the same capacity, there is a possibility of influence of loud sounds even in earlier stages of pregnancy. Because studies on noise effects on the fetus during early pregnancy are lacking, it is impossible to clearly state at what time during pregnancy noise can be harmful to the fetus.

The main limitations of the study concern the exposure classification. Noise exposure was classified through a job–exposure matrix, thereby introducing some misclassification of exposure. Although the matrix accounts for variation in exposure between occupations, it does not account for variation within the occupation (Kromhout and Vermeulen 2001). Neither was it possible to specifically study exposure to low-frequency noise. Although all these factors contribute to misclassification of exposure, it is not likely that this misclassification is differential. In addition, an equal nondifferential misclassification of exposure in each exposure group will lead mainly to an attenuation of the risk in the middle- and high-exposure group because the low-exposure group is so large (80% of the full sample) compared with the middle-exposed (19% of the full sample) and the high-exposed group (1% of the full sample). Even a minor misclassification of the large low-exposure group will lead to a lot of low-exposure mothers to change group to the middle- and high-exposure group, leading to an attenuation of the association in these groups. The increased risk in the high-exposure group > 85 dBA shown in this study exists despite this misclassification, and the true risk estimate might therefore be higher.

To limit the misclassification introduced by the fact that mothers stating the same occupation may be present at the workplace to a various degree, the cohort was subdivided into full-time working mothers, part-time working mothers, and mothers absent from work during pregnancy. Among full-time working mothers, high exposure to occupational noise was associated with an increased risk of hearing dysfunction. An increase in risk was also indicated in the part-time working mothers. There was no increased risk of hearing dysfunction in children whose mothers reported an exposed occupation in the beginning of the pregnancy but were absent from work during pregnancy. The fact that the risk increased with presence at work supports the hypothesis that occupational noise during pregnancy, and not the occupational title as such, is associated with an increased risk of hearing dysfunction.

A statistically significant increase of 5–8% was seen in the intermediate exposure group (75–84 dBA) in the main analysis. The interpretation of this small increase is complex because the class is wide, with a range in exposure of 9 dBA. Most occupations are likely to be found at the lower end of the interval (75–79 dBA), and an effect in the higher end (80–84 dBA) will have a small effect on the estimated risk for the whole intermediate group. Thus, the results for the intermediate group should not be interpreted as a null result or that 75–84 dBA is a safe level for pregnant women to work in. More studies are needed before the highest safe level can be established.

A wide range of hearing dysfunction diagnoses related to noise exposure was selected, mainly because noise-induced hearing dysfunctions in children are difficult to classify and thereby are sometimes classified into the selected “other” categories until a more precise diagnosis can be made. An inclusion of such a mixed group might also contribute to an inclusion of a few cases of hearing dysfunction that are unrelated to noise exposure. However, these “other” diagnoses (not including tinnitus) contributed very few cases (< 10%) and were included only in the dichotomous analyses of all hearing dysfunctions, and should therefore introduce only a minor misclassification of disease. In addition, the misclassification of hearing dysfunction in this study is most likely nondifferential, because occupational exposures to noise during pregnancy is not an established risk factor for hearing dysfunction in children, and it should therefore not make parents more prone to investigate their child’s hearing later in life.

In this study, only hearing dysfunction that was sufficient to prompt an investigation by a specialist could be studied. Thus the analyses are based on few cases, but the low numbers should not be taken as evidence that the problem should be of minor public health relevance. Rather, it is possible that a larger number of cases of mild hearing dysfunction, not identified during childhood or not requiring specialist care, could be induced by occupational noise exposure. This was evident in the earlier three epidemiological studies on occupational noise during pregnancy and hearing dysfunction in children that measured hearing loss for each child by hearing thresholds instead of by diagnoses of hearing dysfunction, and thus also could detect mild hearing loss even in a small sample (Daniel and Laciak 1982; Lalande et al. 1986; Rocha et al. 2007). Even mild hearing loss in children has been associated with increased social and emotional dysfunction among schoolchildren (Bess et al. 1998).

Future studies should investigate the association between different noise frequency intervals and outcome measures of milder hearing dysfunction. The advice given today to pregnant women regarding occupational noise differs substantially among clinics and countries because research on this topic is lacking. This study will therefore provide important information to clinicians and policy makers and will contribute to more accurate advice and guidelines.

## Conclusions

This nationwide population-based study supports the hypothesis that occupational noise exposure during pregnancy is associated with future hearing dysfunction in children. Taken together with previous epidemiological and experimental studies as well as mechanistic data, the available data indicate that pregnant women should not be exposed to high levels of noise at work.

These results need confirmation in further studies. In addition, our results indicate a need to further study the effects of intermediate levels of occupational noise, peak values, and leisure-time exposure, such as rock concerts. Although leisure-time activities are of much shorter duration, the exposure intensity may be very high.
